# Medication-Enhanced Psychotherapy for Posttraumatic Stress Disorder: Recent Findings on Oxytocin’s Involvement in the Neurobiology and Treatment of Posttraumatic Stress Disorder

**DOI:** 10.32872/cpe.3645

**Published:** 2021-12-23

**Authors:** Katrin Preckel, Sebastian Trautmann, Philipp Kanske

**Affiliations:** a Max Planck Institute for Human Cognitive and Brain Sciences, Leipzig, Germany; b Institute of Clinical Psychology and Psychotherapy, Department of Psychology, Medical School Hamburg, Hamburg, Germany; c Clinical Psychology and Behavioral Neuroscience, Faculty of Psychology, Technische Universität Dresden, Dresden, Germany; Philipps-University of Marburg, Marburg, Germany

**Keywords:** PTSD, oxytocin, treatment, medication-enhanced therapy, stress

## Abstract

**Background:**

Traumatic experiences may result in Posttraumatic Stress Disorder (PTSD), which is characterized as an exaggerated fear response that cannot be extinguished over time or in safe environments. What are beneficial psychotherapeutic treatment options for PTSD patients? Can oxytocin (OXT), which is involved in the stress response, and safety learning, ameliorate PTSD symptomatology and enhance psychotherapeutic effects? Here, we will review recent studies regarding OXT’s potential to enhance psychotherapeutic therapies for PTSD treatment.

**Method:**

We conducted a literature review on the neurobiological underpinnings of PTSD especially focusing on OXT’s involvement in the biology and memory formation of PTSD. Furthermore, we researched successful psychotherapeutic treatments for PTSD patients and discuss how OXT may facilitate observed psychotherapeutic effects.

**Results:**

For a relevant proportion of PTSD patients, existing psychotherapies are not beneficial. OXT may be a promising candidate to enhance psychotherapeutic effects, because it dampens responses to stressful events and allows for a faster recovery after stress. On a neural basis, OXT modulates processes that are involved in stress, arousal and memory. OXT effectively counteracts memory impairments caused by stress and facilitates social support seeking which is a key resilience factor for PTSD and which is beneficial in psychotherapeutic settings.

**Conclusion:**

OXT has many characteristics that are promising to positively influence psychotherapy for PTSD patients. It potentially reduces intrusions, but preserves memory of the event itself. Introducing OXT into psychotherapeutic settings may result in better treatment outcomes for PTSD patients. Future research should directly investigate OXT’s effects on PTSD, especially in psychotherapeutic settings.

## Highlights

- Lower endogenous OXT levels after traumatic experiences are associated with developing PTSD.
- OXT administration around the time of the traumatic event may result in fewer intrusive memories.
- Abnormal signaling of the hippocampus and the vmPFC to the amygdala result in hyperactivation of the amygdala in PTSD.
- OXT facilitates social support seeking and safety learning while reducing personal distress.
- OXT’s characteristics are promising to enhance psychotherapeutic treatment for PTSD patients.

Traumatic experiences may result in Posttraumatic Stress Disorder (PTSD), which is characterized as an exaggerated fear response that cannot be extinguished over time or in safe environments. The lifetime prevalence of developing PTSD lies at about 4% (Koenen et al., 2017). This number is relatively low considering that 80% of the general population experience traumatic events during their lifetime. Critical factors that determine if someone develops PTSD after experiencing or witnessing traumatic events include female gender, history of mental disorder (Tortella-Feliu et al., 2019), childhood adversities (McLaughlin et al., 2017), but also individual emotional contagion and empathy (Trautmann et al., 2018). In particular, for witnessed trauma, the ability to distinguish own feelings from that of others is crucial to avoid excessive personal distress and anxiety (Preckel, Kanske, & Singer, 2018). Also, emotion regulation abilities may be indicative of PTSD development after a traumatic experience, as prospective studies on emotion regulation and trauma symptoms show (Bardeen, Kumpula, & Orcutt, 2013; Ehring & Ehlers, 2014).

Treatment approaches for PTSD are not yet sufficiently successful, this is shown by patient drop-out rates, for instance, which are highly variable with rates between 16% to 53.1% (Hatchett & Park, 2003; Lewis, Roberts, Gibson, & Bisson, 2020), depending on how the drop-out rates were defined. Importantly, although trauma‐focused cognitive behavior therapy is the best‐validated treatment for PTSD, it has failed to develop over the past few decades. Importantly, only two‐thirds of PTSD patients respond effectively to this therapy. Besides, the majority of PTSD patients does not take part in evidence‐based treatment, this applies predominately to low‐ and middle‐income countries (Bryant, 2019). This underlines the need for better therapeutic approaches and ongoing research on this topic. Behavioral and medication treatment approaches for PTSD have different strengths and weaknesses (Flanagan & Mitchell, 2019), which makes a combination, of a medication-enhanced psychotherapy approach very promising. Regarding the common symptoms of PTSD such as intrusive memories and flashbacks, avoidance behavior, (negative) changes in cognition and in arousal (American Psychiatric Association, 2013), as well as deficits in social cognition (e.g. empathy, compassion and Theory of Mind) (Couette, Mouchabac, Bourla, Nuss, & Ferreri, 2020; Palgi, Klein, & Shamay-Tsoory, 2016), the neuropeptide and hormone Oxytocin (OXT) may bear relevance for the treatment of PTSD (Palgi, Klein, & Shamay-Tsoory, 2016). OXT may be a relevant treatment enhancer, because it has been found to influence memory (Lee et al., 2015), approach-avoidance behavior (Preckel, Scheele, Kendrick, Maier, & Hurlemann, 2014), social cognition (e.g. emotion recognition) (Schwaiger, Heinrichs, & Kumsta, 2019) and arousal (Rash & Campbell, 2014). Due to OXT’s broad influence on human well-being, it has been introduced as a promising treatment agent for various disorders including PTSD (Koch et al., 2014; Misrani, Tabassum, & Long, 2017; Preckel, Kanske, Singer, Paulus, & Krach, 2016). Furthermore, OXT attenuates the development of PTSD symptoms after trauma exposure in patients with high acute symptomatology (van Zuiden et al., 2017) and is useful as an early preventive intervention (Frijling, 2017). Another study found that OXT was able to reduce PTSD symptoms which were triggered by trauma-script exposure (Sack et al., 2017).

In this update article, we review the latest literature on the relationship of biological underpinnings of PTSD, memory formation and OXT. We investigate the question: What role can/ does OXT play in PTSD symptom development and how might it improve PTSD symptoms?

We start our article by describing the stress physiology and the roles that OXT and cortisol play in it, we then continue by discussing the influence of OXT on the neural circuits of fear conditioning, PTSD and memory and, before summarizing our thoughts, we discuss the potential benefits of OXT as a treatment enhancer for PTSD psychotherapy.

## Stress Physiology and the Roles of Oxytocin and Cortisol

The stress response involves multiple levels, which include cognitive, behavioral and physiological processes. On the physiological level, highly stressful or traumatic experiences, activate the hypothalamic-pituitary-adrenal (HPA) axis as well as the oxytocinergic system (Donadon, Martin-Santos, & Osório, 2018). Activity of the HPA axis and its end-product cortisol facilitate adaption to the faced stressor (e.g. de Kloet, Joëls, & Holsboer, 2005). A typical physiological stressful response involves the following steps: The hypothalamus releases the cortiocotropin-releasing-hormone (CRH) to the pituitary gland, which in turn releases the adrenocorticotropic hormone (ACTH) into systemic circulation. ACTH prompts the adrenal gland to release glucocorticoids such as cortisol. When cortisol levels in the blood increase, this is perceived by brain regions (e.g. hypothalamus) and the release of CRH is stopped to return to homeostasis (Smith & Vale, 2006).

In PTSD patients, the reinstating of homeostasis fails (Yehuda, 2002), resulting in an indiscriminately heightened physiological stress responses (e.g. McFarlane, Atchison, Rafalowicz, & Papay, 1994). Hypocortisolism is often reported in PTSD patients, which might at first sight be counterintuitive. Yet, the downregulation of available cortisol could be an attempt of the body to compensate for exaggerated stress responses (Thaller, Vrkljan, Hotujac, & Thakore, 1999). This compensatory attempt, however, results in a sensitization to the glucocorticoid system (Rohleder, Wolf, & Wolf, 2010), meaning that low concentrations of cortisol are sufficient to induce a fear or stress response. Furthermore, the observed hypocortisolism may be dependent on the type of cortisol measure, because in the cerebrospinal fluid of patients with PTSD, a sustained increase of the corticotropin-releasing hormone was observed (Sherin & Nemeroff, 2011). While hair cortisol levels are commonly reported to be lower in PTSD patients when compared to those of healthy controls (Steudte-Schmiedgen, Kirschbaum, Alexander, & Stalder, 2016; Steudte-Schmiedgen et al., 2015; van Zuiden et al., 2019), but there are contradictory findings (van den Heuvel et al., 2020).

Exogenously administered OXT promotes a faster recovery after the stress response (Heinrichs, Baumgartner, Kirschbaum, & Ehlert, 2003; Kubzansky, Mendes, Appleton, Block, & Adler, 2012), and it attenuates salivary cortisol elevations after a physical stressor (Cardoso, Ellenbogen, Orlando, Bacon, & Joober, 2013). Endogenous OXT levels are frequently measured in the periphery and lower endogenous OXT levels after traumatic experiences are associated with developing PTSD (Donadon, Martin-Santos, & Osório, 2018), even though endogenous OXT levels of individuals who suffer from PTSD and those of healthy controls did not differ (Engel et al., 2019). Interestingly, OXT and cortisol levels are positively correlated when participants were able to anticipate a stressor (Brown, Cardoso, & Ellenbogen, 2016). Anticipation and predictability seem to strongly influence OXT’s action, because also exogenously administered OXT has ambiguous effects on threatening responses which is partly due to the predictability or unpredictability of threatening cues. This means that OXT administration results in anxiogenic effects when threat cues are unpredictable, because defensive responses to unpredictable shocks were significantly increased by OXT (as compared to placebo and vasopression administration), while predictable shocks were not influenced by OXT administration (Grillon et al., 2013). Furthermore, OXT’s effects on anxiety depend on the timing of OXT administration and threat content, because both anxiolytic and anxiogenic effects have been reported (Frijling, 2017; Neumann & Slattery, 2016). Importantly though, in people who experienced moderate emotional trauma, anxiolytic effects of OXT have been found (Donadon et al., 2018). While OXT’s effects on cortisol are diverse, a recent meta-analysis reported that OXT attenuated the cortisol response to a greater extend, when the HPA-axis was strongly activated and this effect was strongest among clinical populations (patients with PTSD, Major Depressive Disorder and Bipolar Disorder) (Cardoso, Kingdon, & Ellenbogen, 2014). These ambiguous findings may be due to the cross-binding ability of OXT and vasopressin, (for more detail see: Preckel & Kanske, 2018). Moreover, OXT enables rapid and flexible adaptation to fear signals in social contexts, which can be advantageous in preventing PTSD; but simultaneously it may elevate vulnerability for interpersonal trauma (Eckstein et al., 2016).

Thus, we assume that the dampening effect of OXT on the HPA axis (Neumann, Krömer, Toschi, & Ebner, 2000) may act on different levels and result in reduced stress responses, thereby eliciting the opposite effects of typical stress tasks such as the Trier Social Stress Test (TSST; Kirschbaum, Pirke, & Hellhammer, 1993). This assumption is grounded in the observation that OXT is associated with faster recovery of the endocrine and the autonomic system after stressful events (Engert et al., 2016), as well as on skin conductance findings which were measured directly after traumatic events and predicted subsequent chronic PTSD development (Hinrichs et al., 2019). Consequently, OXT’s dampening effect on the HPA axis activation may function as a stress-buffer for traumatic events and by buffering stress responses it may prevent the development of chronic PTSD after trauma exposure. The anxiolytic OXT effects may also result in fewer treatment dropouts.

Cortisol, like OXT, has time-sensitive effects on the HPA-axis activity. Activating the HPA-axis by exposing participants to a stress task, for instance the TSST, before they participate in a trauma analogue paradigm (trauma film), results in increased numbers of intrusive memories (in participants who biologically respond to the TSST) as compared to participants who perform a control task (placebo TSST) and are not stressed prior to the trauma film paradigm (Schultebraucks et al., 2019). In contrast, administering cortisol after a trauma results in fewer intrusions (De Quervain, 2006). Outcomes of post-trauma cortisol administration are, however, also somewhat inconclusive, because not all studies report fewer subsequent intrusions (Graebener, Michael, Holz, & Lass-Hennemann, 2017; Ludäscher et al., 2015). Cortisol (here: hydrocortisone) as a treatment enhancer augmented psychological treatment successfully, meaning that prolonged exposure therapy resulted in greater retention when participants received cortisol (Yehuda et al., 2015).

Thus, OXT as well as cortisol are promising agents for medication-tailored treatment for PTSD patients.

## OXT’s Influence on Fear Conditioning, and its Role in PTSD and Memory

To investigate the mechanisms, which underlie PTSD, Pavlovian fear conditioning paradigms are helpful models. In fear conditioning experiments, an aversive stimulus is used as an unconditioned stimulus (US) in order to establish fear as soon as the conditioned stimulus (CS) is presented. In PTSD, one traumatic event is sufficient to establish a CS. The main brain structures that are involved in fear conditioning and PTSD include the amygdala, the hippocampus and the ventromedial prefrontal cortex (vmPFC) (Careaga, Girardi, & Suchecki, 2016; Koenigs & Grafman, 2009). The amygdala is the core structure of fear conditioning (Duvarci & Pare, 2014; Ehrlich et al., 2009) and extinction (Maren, 2011; Myers & Davis, 2002). The different nuclei of the amygdala have specialized roles in the fear learning and extinction processes. The lateral nucleus of the amygdala (LAn) provides the amygdala primarily with input and is important for mediating fear learning via neural plasticity, while the basolateral and basomedial nuclei converge sensory information of the conditioned stimulus and the unconditioned stimulus (Herry & Johansen, 2014). The hippocampus is important for encoding information, and for modulating appropriate emotional responses to potentially fearful stimuli (Acheson, Gresack, & Risbrough, 2012; Lissek & van Meurs, 2015). Furthermore, lower hippocampal activation has been linked to direct memory suppression in healthy participants (Benoit & Anderson, 2012), that can be interpreted as reduced voluntary recall. The vmPFC mediates the extinction of conditioned fear by inhibiting the amygdala (Koenigs et al., 2008).

In PTSD patients, these brain regions differ on a structural and functional level in comparison to healthy controls. For example, reduced hippocampal volume is associated with PTSD development (Gilbertson et al., 2002; Logue et al., 2018; Pitman et al., 2006) as well as being a consequence of stressful experiences (Admon et al., 2013). On a functional level, abnormal hippocampus activation hindered extinction learning in safe contexts (Patel, Spreng, Shin, & Girard, 2012) and reduced top-down regulation to the amygdala which results in enhanced fear conditioning (Rauch, Shin, & Phelps, 2006). A reduction of functional and structural connectivity between the hippocampus and the vmPFC has also been reported (Admon et al., 2013).

The amygdala is also crucially involved in associative learning (LeDoux, 1996; McGaugh, 2000) and its dysfunction may be responsible for increased fear conditioning responses in PTSD patients, which in turn results in stronger memory formation of the traumatic event (= intrusive memories) (Careaga et al., 2016). Also, vmPFC activation is lower and results in decreased top-down regulation of the amygdala (Rauch, Shin, & Phelps, 2006). The hippocampus as well as the vmPFC project to the amygdala and their failure to adequately inhibit amygdala activation causes its hyperactivity which is frequently found in PTSD patients (Hayes, Hayes, & Mikedis, 2012; Liberzon & Abelson, 2016; Patel, Spreng, Shin, & Girard, 2012; Pitman et al., 2012; Shin & Liberzon, 2010). Amygdala hyperactivation is especially pronounced when compared to non-trauma exposed controls, but not necessarily when compared to trauma-exposed controls (Patel et al., 2012), therefore it cannot be ruled out that this mechanism is related to trauma exposure rather than to PTSD (van Wingen, Geuze, Vermetten, & Fernandez, 2011). However, the evidence that amygdala hyperactivation might be causally related to PTSD development, could be shown by previous lesion studies in veterans with and without PTSD (Koenigs & Grafman, 2009). Another lesion study showed that elevated amygdala activation is related to dysfunctional vmPFC activity (Motzkin et al., 2015). Furthermore, PTSD patients (as compared to healthy controls) display an initially increased amygdala response when confronted with trauma-related negative (vs. non-trauma related negative) stimuli (Protopopescu et al., 2005). The elevated amygdala activation may explain the emotional memory quality in PTSD patients, especially, because this activation does not habituate over time (Protopopescu et al., 2005).

Diminished structural connectivity between the amygdala and vmPFC has been found in PTSD patients (Koch et al., 2017). The functional connectivity between these regions, could be increased by OXT (in men with PTSD), thereby reducing amygdala hyperactivity (Koch et al., 2014).

Returning to fear conditioning experiments, administering intranasal OXT before fear conditioning results in faster fear conditioning, (Eckstein et al., 2016) while administration after fear-conditioning and before fear extinction results in better fear extinction and inhibited amygdala activation (Eckstein et al., 2015). Moreover, reduced skin conductance responses to electric shocks after OXT as opposed to placebo administration in human studies support the notion of OXT’s “anti-stress-properties” (Eckstein et al., 2016). Thus, exogenous OXT effects are time sensitive and remain currently inconclusive.

The amygdala is further suggested to mediate influences of medication on memory consolidation (McGaugh, 2000), therefore it may also mediate OXT effects on memory and potentially change the emotional content of memories in PTSD patients. A recent study showed that the severity of childhood trauma exposure (as reported from memory) was related to oxytocin-modulated amygdala responses in patients with PTSD while this was not the case in healthy controls (Flanagan et al., 2019). If OXT has the potential to change the content of memories to turn more positively, this may already result in less hyperactivity of the amygdala, which is strongly influenced by negative valence (Preckel et al., 2019). This is further supported by OXT’s inhibiting effects on the activation of (para-)limbic structures, its facilitating action on cognitive performance and its inhibiting effects on arousal (Lischke, Herpertz, Berger, Domes, & Gamer, 2017; Misrani et al., 2017; Solomon et al., 2018). Animal studies report that exogenous OXT has “anti-stress properties” on hippocampal plasticity and memory (Lee et al., 2015). The hippocampus plays an important role in the negative feedback loop of the HPA-axis (Joseph & Whirledge, 2017) and it is altered in PTSD patients (Schumacher et al., 2019).

Regarding OXT’s effects on memory, earlier studies found that OXT impairs memory recall, the generation of associated target words, or explicit memory (Heinrichs, Meinlschmidt, Wippich, Ehlert, & Hellhammer, 2004). Recent findings, however, suggest that exogenous OXT may also have positive influences on memory. For example, OXT improves safety learning in healthy humans (Eckstein et al., 2019) and animal studies show that OXT effectively counteracts memory impairments caused by stress on a cellular level, thereby preventing memory impairments (Lee et al., 2015). Another animal study found changes in long-term synaptic plasticity in the amygdala (medial nucleus) due to OXT’s action. These oxytocin-induced synaptic changes are strongly related to social recognition memory (Rajamani, Wagner, Grinevich, & Harony-Nicolas, 2018). These findings indicate that OXT may have restoring functions on plasticity related to memory processes.

Furthermore, OXT improves memory performance which is accompanied by increased connectivity between the dorsolateral (dl)PFC and the ACC in traumatized as compared to trauma exposed individuals without PTSD (Flanagan et al., 2018). This is an important finding, because decreased connectivity between the dlPFC and the ACC is described as a maladaptive neural process (= reduced neural processing efficiency) in demanding cognitive tasks. Furthermore, the decrease in connectivity between these brain regions is associated with the trait measure “worry” (as a dimension of anxiety) in healthy individuals (Barker et al., 2018). Regarding the association between worry and PTSD that has been found in previous studies (Blazer, Hughes, & George, 1987), it may be assumed that similar neural maladaptations take place in PTSD and which may be positively influenced by OXT administration. Increased ACC activation after OXT administration has also been reported elsewhere (Preckel, Scheele, Eckstein, Maier, & Hurlemann, 2015). To sum up, OXT positively influences memory on a cellular, neural activation and behavioral level.

Moreover, exogenous OXT was able to improve social behavioral deficits in autism spectrum disorder patients (ASD) via reinstating vmPFC activation, during a social-communication task (Aoki et al., 2015). In male PTSD patients, OXT reinstated diminished connectivity between the amygdala and the vmPFC and in female patients it reestablished increased connectivity between the amygdala and the dorsal anterior cingulate cortex (dACC), accompanied by reduced subjective anxiety and nervousness (Koch et al., 2016b). A recent study showed that OXT dampened amygdala activation in PTSD patients, when they saw emotional faces (regardless of valence), while amygdala activation was increased in trauma-exposed control participants (Koch et al., 2016a). Assuming that OXT’s action in ASD patients is the same as in PTSD patients, as the common action on brain activity suggests, OXT may also improve social and affective functioning in PTSD by restoring vmPFC activation.

## Oxytocin’s Potential Benefit in Psychotherapy for PTSD

Apart from different comorbidities such as depression, anxiety or alcohol abuse, social support is one of the strongest predictors for successful PTSD therapy (Dewar, Paradis, & Fortin, 2020), just like therapeutic alliance (Lantz, 2004). The willingness to share thoughts and emotions is clearly related to perceived social support (Kahn & Cantwell, 2017). As mentioned previously, OXT increases social support seeking and the perception of received social support (Cardoso, Valkanas, Serravalle, & Ellenbogen, 2016) and it also increases the willingness to verbally share one’s emotions with someone else (Lane et al., 2013). This makes it specifically promising for medication-enhanced psychotherapeutic interventions, because it might facilitate emotional disclosure. OXT increases social support seeking and the perception of received social support (Cardoso, Valkanas, Serravalle, & Ellenbogen, 2016) as well as safety learning (Eckstein et al., 2019) in healthy individuals. Assuming that OXT unfolds the same characteristics in PTSD patients, it is likely that OXT can ameliorate PTSD symptoms successfully. In a study on trauma disclosure, OXT alone did not increase the tendency to disclose trauma (Scheele et al., 2019). This might be due to insufficient(ly perceived) social support, because it has also been suggested that the presence of social support might be necessary to elicit prosocial OXT effects (Cardoso et al., 2016), to mention one of many context-dependent OXT effects. Therefore, administering OXT in a psychotherapeutic setting, where social support is available, might result in increased disclosure. Concerning the therapeutic relationship which is important for successful therapy outcomes, increased sensitivity to social reward may result in increased social support seeking and may thus increase the likelihood of a positive psychotherapeutic relationship. Notably, it has been found that anterior insula activation was normalized, during social reward processing, in PTSD patients after OXT administration (Nawijn et al., 2017).

Psychotherapeutic interventions that have been successful in ameliorating PTSD symptoms include eye movement desensitization and reprocessing (Shapiro, 2014) prolonged exposure (Singh, 2019), imagery rescripting and reprocessing therapy (Grunert, Weis, Smucker, & Christianson, 2007), exposure therapy (Paunovic & Ost, 2001) as well as exposure-based cognitive-behavioral group therapy (CBGT) (Schwartze, Barkowski, Strauss, Knaevelsrud, & Rosendahl, 2019). Clinical trials which have investigated OXT’s enhancing effects on different treatment options, revealed that OXT could enhance exposure-therapy in PTSD patients (Flanagan et al., 2019) and patients with arachnophobia (Acheson et al., 2015). A study, which focused on physiological responses to OXT, found one notable difference and that was a higher skin conductance baseline level in the OXT group (Pitman et al., 1993).

Here, we take CBGT as an example to explain how simultaneous OXT administration can enhance psychotherapy.

OXT improves various aspects of social cognition, for example trust (Kosfeld, Heinrichs, Zak, Fischbacher, & Fehr, 2005). Trust is an essential component of psychotherapy that needs to be established first, before the actual therapy can begin (Wampold, 2015). Therefore, if OXT facilitates trust, by decreasing amygdala and dorsal striatum activation as neuroimaging studies were also able to show (Baumgartner, Heinrichs, Vonlanthen, Fischbacher, & Fehr, 2008), it may have beneficial effects on psychotherapeutic outcomes. A recent study on food intake reported that OXT enhances brain activation in areas that govern cognitive control, including the vmPFC (Spetter et al., 2018). Should OXT have the same effects on the vmPFC in PTSD patients, OXT might be particularly beneficial for PTSD patients who take part CBGT. There is a growing literature body which investigates OXT’s potential on psychotherapies for PTSD patients (e.g. Engel et al., 2021; Koch et al., 2014; Koch et al., 2019). Though OXT appears to be a promising candidate to ameliorate PTSD symptoms, especially when combined with psychotherapies, further studies are required to disentangle the exact mechanism of OXT. It is also crucial to find out which PTSD patients can benefit most from OXT-enhanced psychotherapy, because OXT has many person-specific characteristics, ranging from a person’s attachment style to oxytocin receptor gene variations which differentially influence OXT’s action in individuals (Bartz, Zaki, Bolger, & Ochsner, 2011; Olff et al., 2013). Thus, studies with precise designs which combine behavioral, biological, imaging and clinical aspects are required to further address these questions (Giovanna et al., 2020).

## Conclusion and Outlook

In this update paper, we described the mechanisms underlying PTSD by discussing the most recent studies on structural and functional brain changes associated with PTSD, including findings on structural and functional connectivity. We have discussed potential OXT mechanisms of action from the healthy population and ASD patients and related these to mechanisms that are malfunctioning in PTSD patients, thereby building direct implications for OXT’s potential action mechanism. Most importantly, we like to emphasize OXT’s promising characteristics as a psychotherapeutic enhancer. However, there are still uncertainties, which need further investigation. These include the critical aspects of pharmacodynamics and the ideal dosage. It became clear that therapeutic approaches are not yet sufficiently successful in treating PTSD patients, because patients drop out of therapy frequently and some symptoms remain after treatment. OXT remains a promising candidate for medication-tailored PTSD therapy and research on this topic should be continued.
